# Riboflavin Intake Inversely Associated with Cardiovascular-Disease Mortality and Interacting with Folate Intake: Findings from the National Health and Nutrition Examination Survey (NHANES) 2005–2016

**DOI:** 10.3390/nu14245345

**Published:** 2022-12-16

**Authors:** Ming Li, Zumin Shi

**Affiliations:** 1 Division of Health Sciences, University of South Australia, Adelaide, SA 5000, Australia; 2 Human Nutrition Department, College of Health Sciences, QU Health, Qatar University, Doha 2713, Qatar

**Keywords:** riboflavin, all-cause mortality, CVD mortality, association, National Health and Nutrition Examination Survey, adults

## Abstract

The association between intakes of riboflavin and mortality has not been examined intensively in general populations. In this study, 10,480 adults in the 2005–2016 National Health and Nutrition Examination Survey (NHANES) were followed-up until 2019 for their vital status. Riboflavin and folate were assessed by two-day 24 h recall. The date and cause of death were obtained from the US Mortality Registry. The risks of all-cause mortality and cardiovascular disease (CVD) mortality were investigated using a Cox regression analysis. During a mean of 8.5 years follow-up, there were 1214 deaths registered (including 373 deaths from CVD and 302 from cancer). Compared to low level (quartile 1, Q1) of riboflavin intake, the hazard ratios (HRs) (95% confidence interval (CI)) for high level (quartile 4, Q4) were 0.53 (0.31–0.90) for CVD mortality and 0.62 (0.48–0.81) for all-cause mortality. The inverse association between riboflavin intake and CVD mortality was only significant among those with a high intake of folate (*p* for interaction 0.045). Those with a high folate intake (Q4) and low intake of riboflavin (Q1) had the highest risk of CVD mortality (HR 4.38, 95% CI 1.79–10.72), as compared with a high intake of both riboflavin and folate. In conclusion, riboflavin intake was inversely associated with all-cause mortality and CVD mortality, and the association was modified by folate intake.

## 1. Introduction

The most recent report from the World Health Organization (WHO) has shown that three-quarters of all deaths each year are attributable to non-communicable diseases, with cardiovascular diseases (CVD) being the most common cause, followed by cancers, especially in more developed regions [1]. Continuing monitoring the trend of mortality and its cause is of help to evaluate further the burden of health on a population and to investigate the risk for the implementation of health care and interventions. In this regard, epidemiological investigations have been conducted to explore the impact or association between vitamin B and cancer mortality and/or all-cause mortality in various populations. Furthermore, the results are inconsistent. For example, a pooled analysis of 18 randomized controlled trials among patients with renal/CVD/cancers conducted before 2015 showed no association between vitamin B supplementation (folic acid, B6, and B12) and cancer/all-cause mortality [2]. On the other hand, more recent prospective follow-up studies among the general US population have reported either lower serum B12 or dietary B12/B6/folate were associated with a higher risk of CVD mortality/all-cause mortality [3]. In addition, a meta-analysis including both RCTs and follow-up studies until 2018 has indicated the beneficial effect of folate on health outcomes including mortality, CVD, and metabolic-related outcomes [4].

As a vitamin B family member, riboflavin is widely found in both plant- and animal-based foods, including milk, meat, eggs, nuts, enriched flour, and green vegetables. It is a key component of coenzymes involved with the growth of cells, energy production, and the breakdown of fats, steroids, and medications [5]. These biological effects of riboflavin have been comprehensively summarized based on studies for their antioxidant, antiaging, anti-inflammatory, antinociceptive, and anticancer properties [6]. Riboflavin deficiency is associated directly or indirectly with health conditions such as migraine [7], childhood neuropath [8], anemia [9], hypertension [10,11], and cancer [12], to name a few, as synthesized in a recent systematic review [13]. The underlying mechanisms of its effect on each of these health conditions are complex. As for anemia, riboflavin can not only enhance iron absorption but also mobilize iron storage involving ferritin [14].

Studies of public health relevance have related to the importance of riboflavin as a factor in protecting against CVD, cancers, and in vision [15,16]. Yet, its relationship with mortality has not been investigated, unlike other vitamin B family members. For example, data from the Third National Health and Nutrition Examination Survey (NHANESIII) and NHANES 1999–2014 showed higher dietary folate intake reduced all-cause mortality, and CVD mortality by 23% (hazard ratio (HR): 0.77, 95% CI 0.71–0.85) and 41% (HR: 0.59, 95% CI 0.48–0.72) in men and by 14% and 47% in women, respectively [17]. Among participants with a high risk of CVD, a folate supplement reduced the CVD mortality by 44% and all-cause mortality by 28% [18]. Since 1998, folate fortification in cereal grain has been fully implemented in the US [19]. The impact/association of excessive folate on mortality has been investigated, and it has been reported that high red blood cell folates are independently associated with an increased risk of all-cause and CVD mortality among the US national population with diabetes [20], and the relationship is not linear [21]; a similar non-linear relationship was also reported in US populations with chronic kidney diseases [22]. These studies indicate that public health concerns have shifted from the effects of inadequate to excessive folate intake [23]. In addition, studies have indicated that higher folate interacts with other vitamin Bs, exhibiting an adverse effect on cognitive function [24] and colorectal cancer risk [25]. The interaction between riboflavin and folate in relation to mortality has not yet been investigated.

To fill the knowledge gaps, we investigated the association between the dietary intake of riboflavin and mortality in the US general adult population. In addition, we aimed to examine the interaction between riboflavin and folate intake in relation to mortality.

## 2. Method

### 2.1. Study Design and Study Population

This is a follow-up study of adults participating in the 2005–2016 survey of NHANES in the US until the end of 2019 regarding vital status, using linked, de-identified, and publicly available data.

Continuous NHANES is a series of two-year cycle surveys conducted by the Centers for Disease Control and Prevention’s (CDC) National Center for Health Statistics (NCHS) since 1999. The survey samples were selected using a multistage, stratified sampling design and constituted representative samples of the non-institutionalized civilian US population [26]. Participants were interviewed at home and invited for a clinical examination. Data collected in each cycle included the following: demographics, dietary records, examination, laboratory, and questionnaire. Details on the NHANES laboratory/medical technologists’ procedures and anthropometry procedures are described elsewhere [27]. The survey protocol was approved annually by the NCHS Research Ethics Review Board, and all participants provided written informed consent [26]. Data used in this study are publicly accessible on the NHANES website, and no ethical review is required.

**Figure 1:**
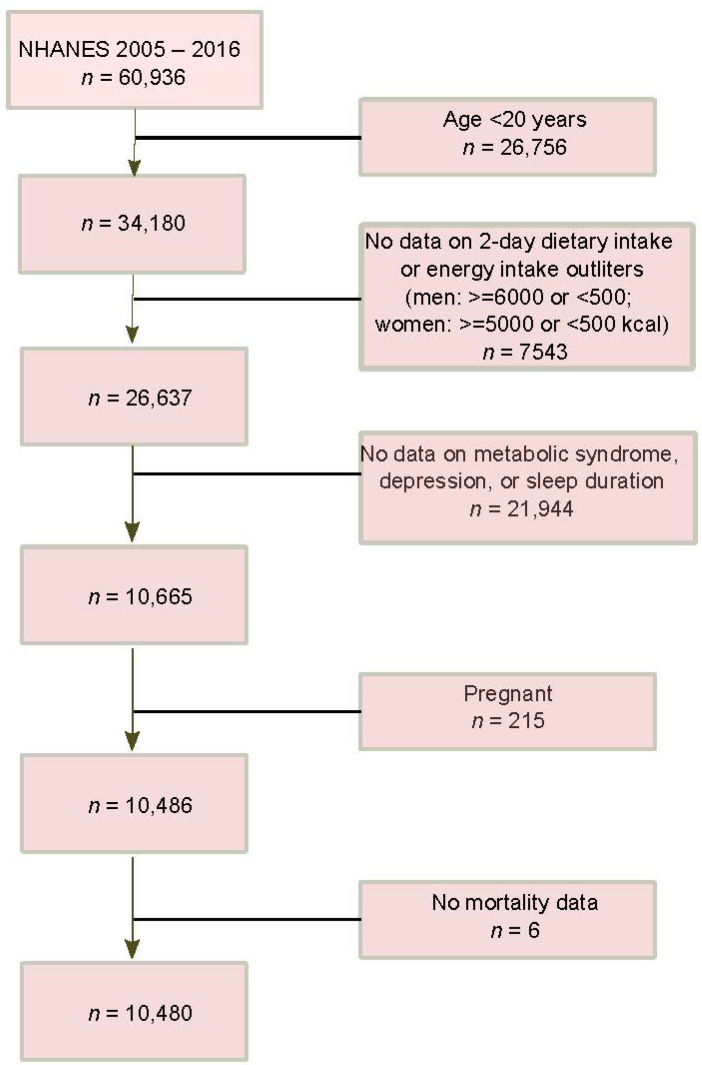
Sample flowchart.

A total of 10,480 adults were eligible for this study based on the following criteria: being aged 20 years and over; having plausible dietary intake records as indicated by daily calories (500–6000 kcal for men, and 500–5000 kcal for women) which were obtained from 2-day 24 h recall records; having health information such as metabolic syndrome, depression, and sleep duration; not being pregnant at the survey; having vital status data (Figure 1).

### 2.2. Study Outcome

The mortality status of participants aged ≥20 years for NHANES participants during 2005–2016 was determined by linking the National Death Index (NDI) [28]. The NDI maintained by NCHS is the nation’s most complete and detailed source of information on mortality in the US. The data include vital status (alive or dead), date of death, and cause of death. Participants who were not deemed to have died as of 31 December 2019, were considered alive. Using the 113 categories of underlying causes of death that were included in the public use files, death was grouped into the following major categories: major cardiovascular diseases (International Classification of Diseases, 10th Revision (ICD-10) I00–I78), malignant neoplasms (ICD-10 C00–C97), and other causes.

### 2.3. Exposure Factors

Dietary intakes of riboflavin and folate were obtained by a 2-day diet recall method. The first day (Day 1) was collected in the Mobile Examination Center (MEC), and the second day (Day 2) was collected by telephone 3 to 10 days later. In total, 87% had 2 days of complete and reliable intakes. Special sample weights were constructed to adjust for the additional dietary interview-specific non-response and the day of week of the dietary intake interview and incorporated in the dietary data files for analysis. The intakes of nutrients from food were evaluated using the United States Department of Agriculture (USDA) Food and Nutrient Database for Dietary Studies (FNDDS), which provided the content of nutrients in each food [29]. The food intake was linked to data from other NHANES components for this study. Detailed information about the dietary interview portion has been published previously [30].

### 2.4. Other Covariates

The study included the following covariates: age; gender; race or ethnicity; education; family income related to poverty; alcohol consumption; energy intake; and self-reported histories of cardiovascular disease (myocardial infarction, stroke, or congestive heart failure), hypertension, metabolic syndrome, depression, and sleep duration. These covariates were collected in the 2005–2016 survey. Smoking status was obtained during the interview, with a non-smoker defined as a participant who had never smoked (had never smoked ≥100 cigarettes during his or her life) or who had quit smoking (had smoked ≥100 cigarettes and was not smoking at the time of inquiry), while a current smoker was defined as having smoked ≥100 cigarettes and was smoking at the time of inquiry. Alcohol consumption was determined from a single 24 h dietary recall and classified as “Yes” or “No”. Leisure time physical activity was classified during the interview as <600, 600–1200, and ≥1200 metabolic equivalents (METS) minutes/week based on self-reported frequency and the duration of participation in moderate and vigorous physical activity during the past 30 days. Weight, height, and blood pressure measures were obtained by the standardized measurement protocol [27].

### 2.5. Statistical Analysis

Population characteristics including percentages and means were compared by quartiles of riboflavin with chi-square tests and ANOVA tests, respectively.

The association between riboflavin and all-cause mortality or CVD mortality was investigated using a Cox regression analysis, adjusting for other covariates. Follow-up time in months was from the date of study entry until the date of death or end of study on 31 December 2019. Schoenfeld residuals were used to test proportionality assumptions which were not deemed to have been violated. For each association analysis, the following models were built: Model 1 adjusted for age, gender, and energy intake based on the unadjusted model; Model 2 further adjusted for education, smoking, alcohol drinking, physical activity, and intake of fat based on Model 1; Model 3 further adjusted for metabolic syndrome, depression, and short sleep based on Model 2. A sensitivity analysis was conducted by incorporating a riboflavin supplement in Model 3.

The multiplicative interaction of riboflavin and each of the other factors with all-cause mortality and CVD morality was tested by introducing a product term of quartiles in the Cox regression model. A stratified analysis by age, gender, ethnicity, smoking, drinking, and folate intake was conducted.

The joint effect of riboflavin and folate on mortality was assessed by a Cox regression analysis using high intake of both folate and riboflavin (quartile 4) as the reference group.

All the analyses were performed using STATA 17.0 (Stata Corporation, College Station, TX, USA). Statistical significance was considered when *p* < 0.05 (two-sided).

## 3. Results

### 3.1. Population Characteristics

A total of 10,480 adults (mean age 50.3 years) participating in surveys during 2005–2016 (6 waves) were included in this study (Figure 1). Among them, 49% were males, 47% were non-Hispanic white, nearly 30% were from low-income families, 20% were smokers, 68% were alcohol drinkers, 40% had lower physical activity, and 35% had shorter sleep duration. In addition, 37% had hypertension, 49% had metabolic syndrome, and 8% had depression.

The mean daily intakes of energy, fat, and protein were 2024.1 (SD 781.9) Kcals, 76.6 (SD 36.3) grams, and 80.2 (SD 34.0) grams, respectively. The mean daily riboflavin intake was 2.1 (SD 1.0) mg ranging from 1.0 (SD 0.2) mg in the lowest quartile (Q1) to 3.4 (SD 1.0) mg in the highest one (Q4). The population’s mean daily folate intake was 397.7 (SD 207.3) microgram or 524.8 (SD 298.3) microgram dietary folate equivalents (DFE). In addition, 26% and 27% participants took riboflavin and folic acid supplements, respectively.

The population characteristics by riboflavin quartiles is shown in Table 1. Compared to the population with the lowest riboflavin intake, those who had the highest intake were significantly younger, males, or non-Hispanic white, or had a higher family income or higher education attainment. They were more likely non-smokers, drinkers, or had a higher physical activity level, and were not short of sleep (all *p* values < 0.001). The prevalence of hypertension, CVD, metabolic syndrome, and depression was lower, but the mean daily intake of macronutrients was higher among those with high riboflavin consumption (all *p* values < 0.01).

### 3.2. Riboflavin Intake and Mortality

By 31 December 2019, with a mean of 8.5 years follow-up (median 8.4, inter-quartile range (IQR) 5.7–11.3), a total of 1214 deaths were registered. Of them, 373 (30.7%) died from CVD, and 302 (24.9%) from cancer.

The weighted overall mortality incident rates (case/1000 person years) for riboflavin quartiles were 12.61, 9.34, 10.20, and 7.00 (*p* < 0.001). Similarly, the CVD mortality incident rate from lower riboflavin intake was significantly higher than the rate from higher intake with the corresponding rates (case/1000 person years) of 3.30, 3.41, 3.14, and 1.84 (*p* < 0.001). However, the cancer mortality incident rate was not different by the riboflavin intake quartiles (*p* = 0.450) (Table 2).

The unadjusted hazard ratio (HR, 95% CI) of all-cause mortality across the quartiles of riboflavin intake was 1.0 (reference), 0.74 (0.59–0.93), 0.80 (0.65–0.99), and 0.61 (0.50–0.75) (*p* for trend < 0.001). The association remained significant after being adjusted for age, gender, and energy in Model 1 with the corresponding HR (95% CI) being 1.00, 0.66 (0.53–0.81), 0.68 (0.55–0.84), and 0.59 (0.46–0.76) (*p* <0.001). The HR did not substantially change after further adjustment for socioeconomic, behavioral, and dietary factors in Model 2 and health risks (metabolic syndrome, depression, and short sleep) in Model 3. Specifically, the fully adjusted HR (95% CI) for all-cause mortality associated with riboflavin intake was 1.0 for the lowest quartile, 0.69 (0.55–0.87) for Q2, 0.74 (0.58–0.93) for Q3, and 0.62 (0.48–0.81) for the highest intake quartile (Model 3, (*p* = 0.002). The strength of association was almost same when the riboflavin supplement was incorporated (*p* = 0.006).

The relative risk of CVD mortality was reduced by 45% for those having the highest riboflavin intake compared to those with the lowest intake (HR: 0.55, 95 CI 0.39–0.77) (*p* = 0.001). The reduced risk remained consistent when adjusted for socioeconomic, behavioral, and dietary factors (Model 1, Model 2), and health conditions (Model 3), with the corresponding HR (95% CI) in Model 3 being 0.53 (0.31–0.90) (*p* = 0.026). The association was not significant when the riboflavin supplement was included (*p* = 0.165).

The unadjusted relative risks for cancer mortality (HR, 95% CI) across the quartiles of riboflavin intake were 1.00 for Q1, 0.73 (0.43–1.26) for Q2, 0.80 (0.50–1.29) for Q3, and 0.81 (0.53–1.24) for Q4 (*p* = 0.450), and the corresponding adjusted HR (9% CI) was 1.00, 0.63 (0.37–1.06), 0.61 (0.37–1.01), and 0.61 (0.36–1.03), respectively (Model 3, *p* = 0.103). The association remained unsignificant when the riboflavin supplement was included (*p* = 0.483).

Female gender, physical activity, and alcohol drinking were inversely associated, but age and smoking were positively associated with all-cause mortality.

The association between riboflavin and CVD mortality did not vary within subgroups by age, or gender, or ethnicity, or smoking, or drinking (Table 3). However, a significant multiplicative interaction between the riboflavin intake and folate intake in relation to CVD mortality was found (*p* for interaction 0.045). Among those with a high intake of folate (Q3 and 4), the HR (95% CI) for CVD was further reduced to 0.48 (0.21–1.09) for Q2, 0.26 (0.11–0.66) for Q3, and 0.20 (0.08–0.50) for Q4, while no such inverse association was found among those with a low intake of folate (Q1 and Q2).

Higher riboflavin intake was associated with the reduced risk of all-cause mortality significantly only in the non-Hispanic white population (HR (95% CI), being 0.61 (0.46–0.81) for Q2, 0.64 (0.49–0.83) for Q3, and 0.53 (0.39–0.70) for Q4 compared to Q1 intake, but not in other ethnic strata. In addition, the association varied by alcohol-drinking status with a significant effect among drinkers, and the corresponding HR (95% CI) for riboflavin intake quartiles was 1 for Q1, 0.51 (0.36–0.72) for Q2, 0.49 (0.35–0.69) for Q3, and 0.39 (0.26–0.58) for Q4 (*p* < 0.001) (Table 4). The association between riboflavin and all-cause mortality seemed lower in the high folate intake subgroup than in the lower intake subgroups, but the difference did not reach the significance level (*p* = 0.111).

**Figure 2:**
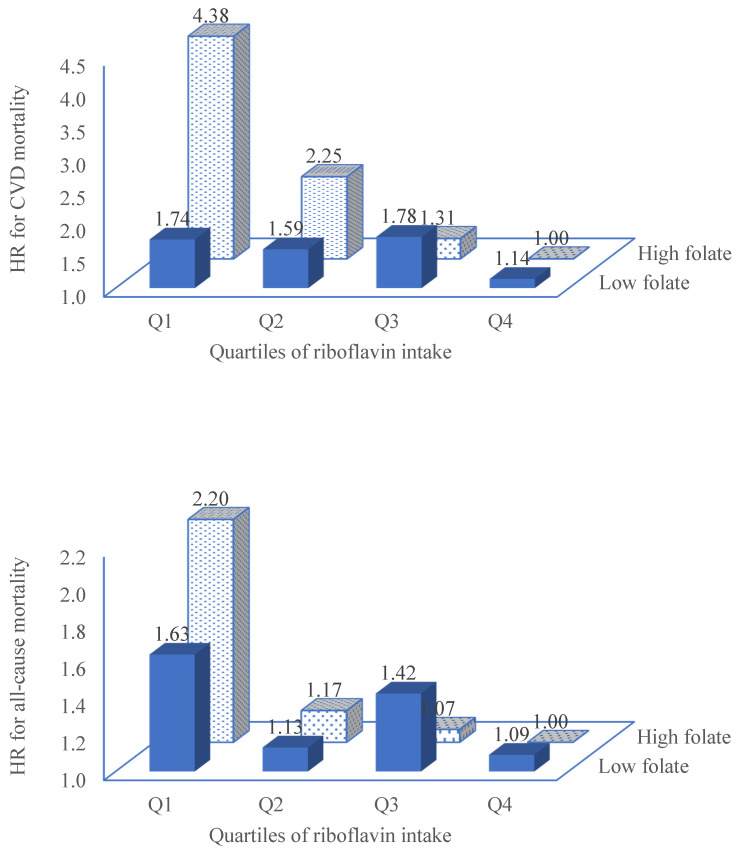
Joint effect of riboflavin and folate intake in relation to CVD mortality and all-cause mortality. Model adjusted for age, gender, race, physical activity, education, smoking, alcohol drinking, intake of energy and fat, metabolic syndrome, depression, and short sleep. Individuals with high intake of folate (quartile 3 and 4) and quartile 4 of riboflavin were used as the reference group. The number of individuals in each group was as follows: high folate (Q1–Q4 of riboflavin, 295, 997, 1703, 2245); low folate (2327, 1622, 916, 375).

The joint effect of riboflavin and folate in relation to CVD mortality is shown in Figure 2. Compared with those with both a high intake of folate and riboflavin (Q4), those with a high intake of folate (Q4) but low riboflavin (Q1) had the highest risk of CVD mortality, especially in women (HR 8.73, 95% CI 2.07–36.85) (Supplementary Table S1). A similar joint effect was found for all-cause mortality.

## 4. Discussion

In these nationally representative, general populations participating NHANES surveys, we have demonstrated that higher consumption of riboflavin was inversely associated with all-cause mortality and CVD mortality. There was a significant interaction between riboflavin and folate intake in relation to CVD mortality. The highest risk of CVD mortality was found among those with a high intake of folate but a low intake of riboflavin.

Our findings of the inverse association between riboflavin and CVD mortality are consistent with current knowledge. Riboflavin plays an important role in folate metabolism as its metabolite, flavin adenine dinucleotide, serves as a cofactor for methylenetetrahydrofolate reductase (MTHFR) [31]. Individuals with the homozygous MTHFR 677 TT genotype have reduced MTHFR activity, which leads to a higher homocysteine concentration [32]. Furthermore, the effect is more marked in individuals with a high folate status. The prevalence of the MTHFR 677 TT genotype ranged from 4 to 26% in populations of European ancestry [33]. People with the MTHFR 677TT genotype had a higher risk of CVD compared with those without this polymorphism [31]. In a clinical trial of 680 healthy adults with a known MTHFR 677C>T genotype, riboflavin lowered blood homocysteine in individuals with the MTHFR 677TT genotype, but different from folate and vitamin B12, riboflavin did not lower homocysteine levels in individuals with other MTHFR genotype groups [34].

An Irish intervention with riboflavin (at dietary levels, 16 mg/d) has reported that riboflavin lowered blood pressure (from 144/87 to 131/80 mmHg) in CVD patients with the TT genotype, but not in the other MTHFR genotype groups [35]. The findings were confirmed in a follow-up study of the original participants [36]. In a large, observational study of 6076 Irish adults, the low or deficient biomarker status of riboflavin was about 30% and exacerbated the genetic risk of hypertension associated with the MTHFR 677TT genotype [37]. In a five-year cohort study of Chinese adults, a higher riboflavin intake was found to be inversely associated with a change in systolic blood pressure [16].

In our study, the inverse association between riboflavin intake and mortality was only significant among those who drank alcohol. It could be explained by the fact that alcoholism increases the risk of riboflavin deficiency [38]. Furthermore, it has been shown that alcohol interferes with folate metabolism and has opposing effects on CVD risks. In the Nurses’ Health Study, heavy drinkers with a lower total folate intake (<180 microgram/day) had the highest risk of major chronic diseases as compared with abstainers with a folate intake of 400–599 microgram/day [39].

There is an ongoing debate on whether the benefits of folic acid fortification outweigh the risk of masking vitamin B12 deficiency [40]. After the mandatory folate fortification in the US, the prevalence of low serum (<10 nmol/L) or red blood cell (<340 nmol/L) folate concentrations was ≤1% [41]. There is concern over the detrimental effect of high folate or folic acid supplements on cognition in older people with low vitamin B12 levels [24]. Our study raised more questions on the long-term health effects of folate fortification. In this population, the mean riboflavin intake was 2.1 mg/day, and the prevalence of the inadequate dietary intake of riboflavin and folate intake was 13.5% and 61%, respectively, using the recommended daily intakes [42]. The increased risk of CVD among those with high folate intake but normal riboflavin intake (Q1) is likely related to high folate level. A joint effect of folate intake and riboflavin intake on colorectal adenomas was reported in a case-control study in the Netherlands, which said that there was a slightly positive association between dietary folate intake and colorectal adenoma risk, especially among those with low vitamin B2 intakes [25].

This is the first assessment of the association between riboflavin and mortality, adding new evidence in the study of the vitamin B family in relation to mortality. The study population was selected with a complex, stratified, multistage probability sampling method. The outcome and study factors were collected based on standardized protocol and robust methodology. The association between riboflavin and mortality was consistent when supplements were incorporated. A series of socioeconomic, behavior, and health factors were collected and able to be adjusted. The sample size was large enough for relatively precise estimates of the association.

Limitations of this study should be noted. Firstly, the long-term habitual intake of riboflavin could not be assessed, in spite of the fact that food intake data were collected using the 2-day 24 h recall method to reduce the within-person variation [43]. Secondly, the study outcome could be a misclassification resulting from data linkage by the probabilistic match method; however, the source data were from the reliable national registry [44]. Thirdly, factors involving food digestion and riboflavin absorption such as gastrointestinal and hepatic functions were not included in this study, and other potential confounding effects of biomarkers could not be assessed. Furthermore, there were no objective measures of riboflavin status in the sample. Serum total folate was only measured in 2007–2008, and 2011–2016. Using the available data, we found that folate intake was significantly associated with serum folate with a correlation coefficient of 0.124 (*p* < 0.001). Finally, the small number of cancer deaths limited further investigation into the association between riboflavin and cancer-specific mortality, since the association varied by cancer type [6,13,15].

In conclusion, by following-up with the national representative adult population in the NHANES surveys during 2005–2016 until the end of 2019, we found that higher riboflavin intake was associated with a decreased risk of all-cause mortality, and specifically, CVD mortality. This protective association from CVD death was strengthened by higher folate intake, while high folate intake could mask the effect of lower riboflavin intake. Further exploration of a balanced diet with an optimal combination of riboflavin and folate intake to prevent excessive CVD mortality is warranted.

## Figures and Tables

**Table 1:** Sample characteristics by quartiles of riboflavin and folate acid intake among adults attending NHANES 2005–2016 (*n* = 10,480).

	Total	Riboflavin Intake Quartiles				
		Q1	Q2	Q3	Q4	*p*
Factors	*n* = 10,480	*n* = 2622	*n* = 2619	*n* = 2619	*n* = 2620	
**Riboflavin (mg/day)**	2.1 (1.0)	1.0 (0.2)	1.6 (0.1)	2.2 (0.2)	3.4 (1.0)	<0.001
**Folate intake (mcg/day)**	397.7 (207.3)	242.8 (101.6)	340.0 (121.6)	426.9 (148.3)	581.2 (251.0)	<0.001
**Folate intake (mcg DFE/day)**	524.8 (298.3)	311.5 (135.8)	440.9 (170.0)	561.9 (216.2)	785.1 (374.5)	<0.001
**Folate intake ≥400 (mcg DFE/day)**	6389 (61.0%)	571 (21.8%)	1406 (53.7%)	2035 (77.7%)	2377 (90.7%)	<0.001
**Protein intake (g/day)**	80.2 (34.0)	53.3 (18.7)	72.1 (22.1)	86.1 (26.1)	109.4 (37.8)	<0.001
**Fat intake (g/day)**	76.7 (36.3)	51.0 (21.1)	68.8 (25.8)	83.3 (30.7)	103.6 (41.5)	<0.001
**Energy intake (kcal/day)**	2024.1 (781.9)	1408.1 (451.3)	1825.6 (528.3)	2173.2 (602.7)	2689.8 (845.7)	<0.001
**Age (years)**	50.3 (17.6)	50.0 (18.1)	51.0 (17.7)	51.3 (17.5)	48.9 (17.0)	<0.001
**Gender**						<0.001
Men	5143 (49.1%)	899 (34.3%)	1058 (40.4%)	1400 (53.5%)	1786 (68.2%)	
Women	5337 (50.9%)	1723 (65.7%)	1561 (59.6%)	1219 (46.5%)	834 (31.8%)	
**Race**						<0.001
NH White	4972 (47.4%)	858 (32.7%)	1128 (43.1%)	1357 (51.8%)	1629 (62.2%)	
NH Black	2001 (19.1%)	755 (28.8%)	571 (21.8%)	389 (14.9%)	286 (10.9%)	
Mex American	1587 (15.1%)	436 (16.6%)	382 (14.6%)	409 (15.6%)	360 (13.7%)	
Other race/ethnicity	1920 (18.3%)	573 (21.9%)	538 (20.5%)	464 (17.7%)	345 (13.2%)	
**Education**						<0.001
<11 grade	2494 (23.8%)	822 (31.4%)	633 (24.2%)	545 (20.8%)	494 (18.9%)	
HS displ or GED	2400 (22.9%)	659 (25.2%)	559 (21.4%)	618 (23.6%)	564 (21.5%)	
Some college	3042 (29.0%)	702 (26.8%)	813 (31.1%)	715 (27.3%)	812 (31.0%)	
>college	2536 (24.2%)	436 (16.6%)	610 (23.3%)	741 (28.3%)	749 (28.6%)	
**Ratio of family income to poverty**						<0.001
<1.30	2904 (29.9%)	890 (37.2%)	700 (29.1%)	645 (26.5%)	669 (27.1%)	
1.3–3.5	3717 (38.3%)	966 (40.4%)	978 (40.6%)	912 (37.5%)	861 (34.9%)	
>3.5	3083 (31.8%)	537 (22.4%)	730 (30.3%)	876 (36.0%)	940 (38.1%)	
**Smoking**						<0.001
Never	5690 (54.3%)	1505 (57.4%)	1512 (57.8%)	1405 (53.6%)	1268 (48.4%)	
Former	2697 (25.7%)	575 (21.9%)	613 (23.4%)	722 (27.6%)	787 (30.0%)	
Current smoker	2089 (19.9%)	540 (20.6%)	492 (18.8%)	492 (18.8%)	565 (21.6%)	
**Alcohol drinking**						<0.001
No	1937 (18.5%)	526 (20.1%)	497 (19.0%)	454 (17.3%)	460 (17.6%)	
Yes	7139 (68.1%)	1597 (60.9%)	1747 (66.7%)	1858 (70.9%)	1937 (73.9%)	
Missing	1404 (13.4%)	499 (19.0%)	375 (14.3%)	307 (11.7%)	223 (8.5%)	
**BMI (kg/m^2^)**	29.1 (6.7)	29.3 (6.9)	29.4 (7.1)	28.8 (6.3)	28.8 (6.5)	<0.001
**Leisure time physical activity (MET/Wk)**						<0.001
<600	4149 (39.6%)	1226 (46.8%)	1106 (42.2%)	1002 (38.3%)	815 (31.1%)	
600–1200	1218 (11.6%)	264 (10.1%)	332 (12.7%)	326 (12.5%)	296 (11.3%)	
≥1200	5112 (48.8%)	1132 (43.2%)	1181 (45.1%)	1290 (49.3%)	1509 (57.6%)	
**Hypertension**	3871 (37.0%)	1046 (40.0%)	1041 (39.8%)	952 (36.4%)	832 (31.8%)	<0.001
**Metabolic syndrome**	5123 (48.9%)	1333 (50.8%)	1326 (50.6%)	1288 (49.2%)	1176 (44.9%)	<0.001
**Depression**	842 (8.0%)	252 (9.6%)	201 (7.7%)	202 (7.7%)	187 (7.1%)	0.006
**Short sleep**	3657 (34.9%)	972 (37.1%)	941 (35.9%)	868 (33.1%)	876 (33.4%)	0.005
**CVD**	1167 (11.1%)	330 (12.6%)	311 (11.9%)	281 (10.7%)	245 (9.4%)	0.001

Data are presented as mean (SD) for continuous measures, and *n* (%) for categorical measures. *p* value from chi-square test for categorical variables and ANOVA for continuous variables.

**Table 2:** Association between riboflavin intake and mortality among adults attending NHANES 2005–2016.

	Quartiles of Riboflavin Intake				
Study Outcomes	Q1	Q2	Q3	Q4	*p*
**All-cause mortality**					
Number of total deaths	314	303	312	285	
Incidence rate (per 1000 person years)	12.61	9.34	10.20	7.00	
Unadjusted	1.00	0.74 (0.59–0.93)	0.80 (0.65–0.99)	0.61 (0.50–0.75)	<0.001
Model 1	1.00	0.66 (0.53–0.81)	0.68 (0.55–0.84)	0.59 (0.46–0.76)	<0.001
Model 2	1.00	0.69 (0.55–0.87)	0.74 (0.58–0.94)	0.62 (0.48–0.80)	0.002
Model 3	1.00	0.69 (0.55–0.87)	0.74 (0.58–0.93)	0.62 (0.48–0.81)	0.002
Sensitivity analysis	1.00	0.69 (0.52–0.92)	0.73 (0.56–0.96)	0.65 (0.50–0.84)	0.006
**CVD mortality**					
Number of deaths from CVD	95	100	106	72	
Incidence rate (per 1000 person years)	3.30	3.41	3.14	1.84	
Unadjusted	1.00	1.02 (0.75–1.39)	0.93 (0.68–1.29)	0.55 (0.39–0.77)	0.001
Model 1	1.00	0.85 (0.59–1.21)	0.69 (0.45–1.08)	0.44 (0.27–0.73)	0.003
Model 2	1.00	0.92 (0.63–1.35)	0.79 (0.49–1.26)	0.52 (0.30–0.90)	0.027
Model 3	1.00	0.94 (0.64–1.36)	0.78 (0.49–1.25)	0.53 (0.31–0.90)	0.026
Sensitivity analysis	1.00	1.00 (0.65–1.53)	0.81 (0.46–1.43)	0.72 (0.43–1.20)	0.165
**Cancer mortality**					
Number of deaths from cancer	74	71	69	88	
Incidence rate (per 1000 person years)	3.04	2.18	2.43	2.45	
Unadjusted	1.00	0.73 (0.43–1.26)	0.80 (0.50–1.29)	0.81 (0.53–1.24)	0.450
Model 1	1.00	0.61 (0.36–1.04)	0.59 (0.36–0.96)	0.62 (0.39–1.00)	0.085
Model 2	1.00	0.62 (0.37–1.06)	0.61 (0.37–1.00)	0.61 (0.36–1.02)	0.095
Model 3	1.00	0.63 (0.37–1.06)	0.61 (0.37–1.01)	0.61 (0.36–1.03)	0.103
Sensitivity analysis	1.00	0.51 (0.27–0.98)	0.88 (0.51–1.51)	0.67 (0.38–1.17)	0.483

Model 1 adjusted for age, gender, and energy intake; Model 2 further adjusted for physical activity, education, smoking, alcohol drinking, and intake of fat; Model 3 further adjusted for metabolic syndrome, depression, and short sleep; sensitivity analysis: Model 3 including riboflavin supplement. *p* value from Cox regression analysis.

**Table 3:** Subgroup analyses of the association between quartiles of riboflavin intake and CVD mortality.

	Quartiles of Riboflavin Intake					
Factors	Q1	Q2	Q3	Q4		*p*
**Age (years)**						0.268
20–39	1.00	0.28 (0.03–2.52)	0.10 (0.01–1.16)	0.16 (0.04–0.56)	0.008	
40–59	1.00	0.51 (0.14–1.84)	0.35 (0.08–1.53)	0.31 (0.05–1.82)	0.206	
60+	1.00	1.08 (0.73–1.59)	1.02 (0.64–1.63)	0.66 (0.39–1.11)	0.142	
**Gender**						0.185
Men	1.00	1.21 (0.62–2.34)	0.75 (0.37–1.53)	0.61 (0.30–1.23)	0.055	
Women	1.00	0.80 (0.46–1.39)	1.01 (0.47–2.18)	0.43 (0.15–1.20)	0.351	
**Race**						0.290
NH White	1.00	0.92 (0.55–1.54)	0.73 (0.43–1.23)	0.44 (0.23–0.82)	0.008	
NH Black	1.00	0.55 (0.28–1.10)	0.91 (0.41–2.02)	0.90 (0.40–2.03)	0.866	
Mex American	1.00	1.32 (0.33–5.28)	0.97 (0.19–5.06)	1.13 (0.29–4.40)	0.923	
Other race/ethn	1.00	0.71 (0.19–2.73)	0.55 (0.12–2.43)	0.62 (0.09–4.01)	0.582	
**Smoking**						0.541
Never	1.00	0.89 (0.54–1.49)	0.76 (0.38–1.56)	0.48 (0.21–1.08)	0.108	
Former	1.00	1.14 (0.57–2.25)	1.04 (0.47–2.34)	0.65 (0.28–1.55)	0.314	
Current smoker	1.00	0.64 (0.19–2.09)	0.66 (0.19–2.31)	0.48 (0.13–1.81)	0.312	
**Alcohol drinking**						0.849
No	1.00	0.93 (0.49–1.78)	0.77 (0.30–1.95)	0.60 (0.23–1.57)	0.308	
Yes	1.00	0.90 (0.49–1.67)	0.65 (0.36–1.18)	0.39 (0.18–0.84)	0.009	
Missing	1.00	0.86 (0.33–2.25)	1.29 (0.40–4.19)	1.19 (0.24–5.95)	0.722	
**Dietary folate intake (DFE, quartiles)**						0.045
Q1–Q2	1.00	0.95 (0.59–1.53)	1.22 (0.65–2.28)	0.69 (0.30–1.56)	0.940	
Q3–Q4	1.00	0.48 (0.21–1.09)	0.26 (0.11–0.66)	0.20 (0.08–0.50)	0.003	

Model adjusted for age, gender, race, physical activity, education, smoking, alcohol drinking, and intake of energy and fat. Stratification variables were not adjusted in the corresponding model. *p* value from Cox regression analysis of interaction term of the listed variable and riboflavin.

**Table 4:** Subgroup analyses of the association between quartiles of riboflavin intake and all-cause mortality.

	Quartiles of Riboflavin Intake					
Factors	Q1	Q2	Q3	Q4		*p*
**Age group**						0.467
20–39	1.00	0.54 (0.14–2.04)	1.36 (0.28–6.69)	0.57 (0.09–3.70)	0.765	
40–59	1.00	0.40 (0.19–0.83)	0.59 (0.31–1.11)	0.51 (0.26–1.03)	0.165	
60+	1.00	0.77 (0.61–0.97)	0.76 (0.60–0.95)	0.67 (0.50–0.89)	0.009	
**Gender**						0.776
Men	1.00	0.75 (0.53–1.07)	0.75 (0.53–1.07)	0.60 (0.41–0.88)	0.020	
Women	1.00	0.64 (0.47–0.86)	0.73 (0.51–1.03)	0.64 (0.45–0.91)	0.025	
**Race/ethnicity**						0.039
NH White	1.00	0.61 (0.46–0.81)	0.64 (0.49–0.83)	0.53 (0.39–0.70)	<0.001	
NH Black	1.00	0.95 (0.60–1.51)	0.98 (0.55–1.73)	1.17 (0.62–2.21)	0.799	
Mex American	1.00	0.80 (0.44–1.46)	1.05 (0.47–2.34)	0.73 (0.26–2.06)	0.796	
Other	1.00	0.94 (0.39–2.26)	2.10 (0.66–6.70)	1.76 (0.47–6.53)	0.230	
**Smoking**						0.223
Never	1.00	0.64 (0.47–0.85)	0.72 (0.48–1.08)	0.69 (0.45–1.06)	0.164	
Former	1.00	0.72 (0.52–1.01)	0.70 (0.46–1.06)	0.58 (0.36–0.95)	0.043	
**Alcohol drinking (past 12 months)**						0.010
No	1.00	1.06 (0.73–1.54)	1.25 (0.82–1.89)	1.16 (0.75–1.78)	0.443	
Yes	1.00	0.51 (0.36–0.72)	0.49 (0.35–0.69)	0.39 (0.26–0.58)	<0.001	
Missing	1.00	0.67 (0.38–1.18)	1.05 (0.64–1.72)	1.14 (0.57–2.27)	0.586	
Current smoker	1.00	0.81 (0.46–1.45)	0.99 (0.57–1.72)	0.71 (0.40–1.28)	0.338	
**Folate intake (DFE, quartiles)**						0.111
Q1–Q2	1.00	0.71 (0.54–0.93)	0.93 (0.67–1.29)	0.71 (0.44–1.16)	0.278	
Q3–Q4	1.00	0.48 (0.29–0.80)	0.43 (0.25–0.73)	0.40 (0.24–0.69)	0.047	

Model adjusted for age, gender, race, physical activity, education, smoking, alcohol drinking, and intake of energy and fat. Stratification variables were not adjusted in the corresponding model. *p* from Cox regression analysis of interaction term of the listed variable and riboflavin.

## Data Availability

The data are publicly available online (https://wwwn.cdc.gov/nchs/nhanes/Default.aspx; accessed on 22 July 2022).

## Supplementary Materials

The following supporting information can be downloaded at: https://www.mdpi.com/article/10.3390/nu14245345/s1, Table S1: Joint effect of riboflavin intake and folate intake in relation to CVD mortality and all-cause mortality.
