# Supplementary material for: Riboflavin Intake Inversely Associated with Cardiovascular-Disease Mortality and Interacting with Folate Intake: Findings from the National Health and Nutrition Examination Survey (NHANES) 2005–2016
Source: Nutrients. 2022 Dec 16;14(24):5345. doi: 10.3390/nu14245345 (PMC9785396; doi:10.3390/nu14245345)
Supplement: Supplementary file 1 [file nutrients-14-05345-s001.zip › nutrients-2088154-supplementary.pdf]

Supplementary Table S1. Joint effect of riboflavin intake and folate intake in relation to CVD mortality and all-cause mortality.

Study Outcomes		Both Genders		Men		Women	
		HR (95% CI)	*p*-value	HR (95% CI)	*p*-value	HR (95% CI)	*p*-value
**All-cause mortality**							
Low folate intake	Riboflavin intake						
	Q1	1.63 (1.22–2.18)	0.001	1.69 (1.10–2.59)	0.018	1.56 (1.08–2.26)	0.017
	Q2	1.13 (0.85–1.51)	0.402	1.31 (0.89–1.93)	0.176	0.99 (0.70–1.38)	0.943
	Q3	1.42 (1.06–1.90)	0.021	1.33 (0.87–2.04)	0.192	1.43 (0.99–2.07)	0.055
	Q4	1.09 (0.75–1.57)	0.649	1.19 (0.78–1.81)	0.420	0.96 (0.49–1.90)	0.910
High folate intake	Riboflavin intake						
	Q1	2.20 (1.27–3.82)	0.006	1.82 (0.84–3.96)	0.129	2.56 (1.13-5.83)	0.025
	Q2	1.17 (0.84–1.62)	0.349	1.23 (0.71–2.12)	0.464	1.08 (0.73–1.61)	0.684
	Q3	1.07 (0.86–1.33)	0.562	1.24 (0.95–1.62)	0.110	0.89 (0.58–1.39)	0.618
	Q4	1.00		1.00		1.00	
**CVD mortality**							
Low folate intake	Riboflavin intake						
	Q1	1.74 (0.93–3.28)	0.083	1.53 (0.68–3.44)	0.297	2.29 (0.77–6.78)	0.135
	Q2	1.59 (0.93–2.72)	0.089	1.97 (1.04–3.74)	0.038	1.67 (0.63–4.42)	0.298
	Q3	1.78 (1.14–2.81)	0.013	1.38 (0.82–2.34)	0.223	2.96 (1.06–8.27)	0.039
	Q4	1.14 (0.45–2.85)	0.784	1.18 (0.44-3.15)	0.743	1.41 (0.28–7.12)	0.672
High folate intake	Riboflavin intake						
	Q1	4.38 (1.79–10.72)	0.001	2.68 (0.67–10.67)	0.160	8.73 (2.07–36.85)	0.004
	Q2	2.25 (1.21–4.19)	0.011	2.18 (0.89–5.33)	0.088	3.06 (1.14–8.21)	0.026
	Q3	1.31 (0.89–1.93)	0.175	1.15 (0.70–1.89)	0.577	2.19 (1.14–4.19)	0.019
	Q4	1.00		1.00		1.00	

CVD: cardiovascular diseases; Model adjusted for age, gender, race, physical activity, education, smoking, alcohol drinking, intake of energy and fat, metabolic syndrome, depression, and short sleep. Individuals with high intake of folate (quartile 3 and 4) and quartile 4 of riboflavin were used as the reference group. The number of individuals in each group was: high folate (Q1-Q4 of riboflavin, 295, 997, 1703, 2245); low folate (2327, 1622, 916, 375).
